# Self-directed or therapist-led parent training for children with attention deficit hyperactivity disorder? A randomized controlled non-inferiority pilot trial

**DOI:** 10.1016/j.invent.2019.100262

**Published:** 2019-08-08

**Authors:** Simone Breider, Annelies de Bildt, Maaike H. Nauta, Pieter J. Hoekstra, Barbara J. van den Hoofdakker

**Affiliations:** a University of Groningen, University Medical Center Groningen, Department of Child and Adolescent Psychiatry, Lübeckweg 2, 9723 HE Groningen, the Netherlands; b University of Groningen, Department of Clinical Psychology and Experimental Psychopathology, Grote Kruisstraat 2/1, 9712 TS Groningen, the Netherlands

**Keywords:** Online, Blended, Face-to-face, Non-inferiority, Attrition, Behavior problems

## Abstract

**Background and objectives:**

Therapist-led behavioral parent training is a well-established treatment for behavior problems in children with attention-deficit/hyperactivity disorder (ADHD). However, parental attrition is high; self-directed forms of parent training may be a promising alternative. To date, no studies have compared these two forms of parent training in referred children with ADHD. The objectives of this pilot study were to examine the non-inferiority of a blended parent training (i.e. online program + supportive therapist contact) in comparison to its therapist-led equivalent (i.e. face-to-face parent training) regarding effects on behavioral problems, and to compare attrition rates, parental satisfaction, and therapist-time between both treatments.

**Methods:**

21 school-aged children with ADHD and behavioral problems, who had been referred to an outpatient mental health clinic, were randomized to blended (*n* = 11) or face-to-face (*n* = 10) parent training. Behavior problems were measured with the Child Behavior Checklist. Treatment completers and dropouts were included in the analyses.

**Results and conclusions:**

Blended parent training was not found to be non-inferior to face-to-face parent training in the reduction of behavior problems. Parents in the blended condition dropped out of treatment significantly earlier than parents in the face-to-face condition and were less satisfied. Therapists in the blended condition spent significantly less time on parent training than therapists in the face-to-face condition.

## Highlights

• This pilot study compared self-directed and therapist-led parent training for children with ADHD and behavior problems
• Self-directed parent training was not non-inferior in the reduction of behavior problems, compared to therapist-led training
• Parents receiving self-directed parent training dropped out of treatment earlier and were less satisfied

## 1 Introduction

Behavioral parent training is a well-established treatment for preschool and elementary school-aged children with attention/deficit-hyperactivity disorder (ADHD; Evans et al., 2018). It has been shown that individual training programs as well as group formats effectively reduce behavioral problems in children with ADHD (Coates et al., 2015; Daley et al., 2014; Lundahl et al., 2006). However, dropout rates may be high, which has been associated with suboptimal treatment outcome (Abrahamse et al., 2016; Boggs et al., 2005). In a review of 262 studies concerning behavioral parent training for parents of children aged 2 to 12 with ADHD, oppositional defiant disorder, conduct disorder, or conduct behavioral problems, it was estimated that 25% of the parents chose not to participate at all in parent training and 26% of the participants dropped out before or during the treatment. Moreover, participants who did start parent training attended only 73% of sessions on average (Chacko et al., 2016). Although engagement and attendance rates varied widely between the studies in this review, the high overall numbers point to the importance of investigating the reasons for dropout and finding ways to reduce it.

Particular barriers for starting and attending parent training programs have shown to be the considerable amounts of time and resources parents have to invest (Koerting et al., 2013; Mytton et al., 2014; Owens et al., 2002). The treatment often contains ten or more clinic-based sessions in which parents learn parenting skills which they are expected to implement and practice at home (Coates et al., 2015). Parents have to organize transportation, travel to the treatment location, and find a suitable time in accordance with their, often busy, daily routine. Furthermore, the availability of the treatment (i.e. long waiting lists) can be a barrier for participation (Koerting et al., 2013). Also, parent training is a time-consuming treatment for therapists in clinical practice, where recourses are often limited. This may also limit the availability of parent training for parents.

To overcome barriers of parent training programs, self-directed parenting interventions (e.g. programs using video, audio, reading material, and/or online learning programs, with or without therapist support) are increasingly being considered as an alternative to therapist-led programs (e.g. Baumel et al., 2016; Corralejo and Domenech Rodríguez, 2018; McGoron and Ondersma, 2015; Montgomery et al., 2006; Tarver et al., 2014). However, it is largely unknown whether these programs are as effective as therapist-led programs and whether they lead to less parental attrition, higher parental satisfaction and less therapist time in children with ADHD. Only few studies have directly compared both forms of parent training specifically in children with ADHD. Furthermore, little is known about self-directed interventions in samples of children who have been referred to a mental health service, whereas most studies have used samples of children recruited from the general population. However, referred children with ADHD who need parent training may be more complex than non-referred children. Therefore, it is important to investigate self-directed interventions in referred samples.

Meta-analytic studies on self-directed parenting interventions have shown moderate to large effects on children's behavior problems compared to waitlist control groups (Baumel et al., 2016; Tarver et al., 2014). The studies in these meta-analyses included interventions with or without therapist support (e.g. video-tape training, online parent training, and workbook training) for (mostly recruited) children with disruptive behavior problems. Only one of the studies that were included in these meta-analyses was conducted specifically in children with ADHD (Daley and O'Brien, 2013). This study showed that self-directed parent training was effective on the reduction of ADHD symptoms in 4 to 11 year old referred children, compared to a waitlist control group. Parents in this study attended a face-to-face introduction, after which they received a written manual in which they worked for six weeks while receiving weekly phone calls (i.e. to remind and monitor, non-therapeutic). Another study (not included in the meta-analyses of Baumel et al. (2016) and Tarver et al. (2014)) demonstrated that online parent training was effective in reducing behavioral problems in recruited preschool children with ADHD symptoms (Franke et al., 2016). After receiving online parent training, consisting of eight online modules and two telephone consultations, mothers in this study reported improved child behavior in contrast with a delayed intervention group.

There are also few studies that have compared self-directed parent training to their therapist-led equivalents. In the above mentioned meta-analysis (Tarver et al., 2014) four of such studies, focusing on children with either oppositional or conduct problems, were included. No significant differences were found in the decrease of externalizing problems between the self-directed and therapist-led parent training formats. Another meta-analysis (Lundahl et al., 2006) examined treatment modality as a moderator for the effect of parent training programs targeting disruptive child behaviors. Across studies, no significant difference was found between the effect of self-directed and therapist-led interventions on child behavior. To our knowledge, only one study investigated the effect of self-directed and therapist-led parent training in relation to ADHD (DuPaul et al., 2018). This study used a randomized controlled pilot with recruited preschool children at risk for ADHD and demonstrated that both a therapist-led group parent training and an online parent training (consisting of a first face-to-face session, ten online sessions, and weekly telephone contact) resulted in improved child behavior in comparison to a waitlist control group. In general, self-directed parenting interventions differ in the amount of therapist support. Studies have shown that therapist support (e.g. telephone contact, online contact, face-to-face appointments), can positively affect the effects of these programs (Day and Sanders, 2018; O'Brien and Daley, 2011; Tarver et al., 2014; Webster-Stratton, 1990).

Findings regarding attrition rates of self-directed parenting interventions in general are mixed, with some studies reporting low attrition rates and other studies reporting attrition rates similar to the rates in therapist-led interventions (Breitenstein et al., 2014; Hall and Bierman, 2015). For ADHD specific, completion rates range from 55% (Franke et al., 2016), to 80% (DuPaul et al., 2018), or even 87.5% (Daley and O'Brien, 2013). Therapist support may be important to enhance completion rates, as in a study an online parenting program that included parents of children aged 1 to 8 year with behavioral concerns (i.e. symptoms of conduct disorder, oppositional defiant disorder, or ADHD), parents who received telephone support completed more training modules and finished the program more often than parents who did not receive support (Day and Sanders, 2018).

Findings concerning parental satisfaction about self-directed are mixed as well. Some studies reported that parents of children with elevated levels of child behavior problems or ADHD symptoms were satisfied about self-directed parenting programs (Franke et al., 2016; Markie-Dadds and Sanders, 2006; Metzler et al., 2012; Sanders et al., 2012; Stewart and Carlson, 2010). However, parental satisfaction was lower in self-directed than therapist-led parent training in a sample of children at risk for ADHD (DuPaul et al., 2018) and in a sample of children with early onset conduct problems (Sanders et al., 2000). Therapist support in self-directed parenting interventions may also positively affect parental satisfaction (Day and Sanders, 2018; Rabbitt et al., 2016).

Although the findings of above studies point towards several benefits of self-directed parenting interventions (with therapist support), only one study investigated the costs (i.e. based on therapist time that was mainly needed for providing online feedback to the parents and salary) of an online parent training and estimated it to be three times less expensive than a therapist-led group parent training (Enebrink et al., 2012).

The current study was a randomized controlled pilot trial comparing two types of behavioral parent training for children with ADHD and behavior problems who have been referred to a mental health clinic: an individual self-directed online parent training program with online therapist feedback and a few supportive face-to-face parent-therapist contacts (i.e. blended parent training) and its therapist-led equivalent (i.e. individual face-to-face clinic-based parent training). The face-to-face parent training (group format) has been found effective in reducing behavior problems in 4 to 12 year old children with ADHD, compared to a control group (Van den Hoofdakker et al., 2007) and to improve behavior problems over time in preschool children with ADHD (individual or group format; Van der Veen-Mulders et al., 2018). In the current study, we aimed to examine whether the effect of the blended parent training was non-inferior compared to the face-to-face parent training with regard to the reduction of child behavior problems. Furthermore, we aimed to investigate whether the blended parent training effectively addressed barriers of therapist-led parent training regarding attrition rates, parental satisfaction, and time spent by the therapists between the two parent training formats.

## 2 Material and methods

### 2.1 Recruitment

We aimed to include twenty children from an outpatient mental health clinic in the Netherlands based on the following criteria: the child 1) had a DSM-IV-TR (American Psychiatric Association, 2000) or DSM-5 (American Psychiatric Association, 2013) diagnosis of ADHD, as assessed by an experienced psychologist with postmaster degree, or child- and adolescent psychiatrist, after referral to the clinic; 2) was between 4 and 12 years old; 3) had an IQ higher than 70; 4) took no psychotropic medication or was on a stable dose for at least six weeks before entering the study, and the prescribing clinician was not expecting changes in dose or agent; 5) at least one parent experienced behavior problems at home, as assessed by a list of target behaviors (see Van den Hoofdakker et al., 2007); 6) both parents were willing to participate in the parent training; and 7) parents had a laptop or PC at their disposal. Parents who had participated in a behavioral parent training in the year prior to the study were excluded, as were families that needed immediate intervention (e.g. families in which one of the parents was experiencing acute psychiatric problems or in which the safety of the child could not be guaranteed). No restrictions were placed on children's comorbid diagnoses, as assessed from the electronic patient records. Children and their parents were referred to the researchers by their clinician. Ethical approval was received from the Medical Ethics Review Committee of the University Medical Center Groningen. Inclusion took place from January 2016 to June 2017. Informed consent was obtained from the parents of the included children. This trial has been registered in the Netherlands Trial Register (see https://www.trialregister.nl/trial/6092; study acronym: ATHENE).

### 2.2 Study design

The current study was a pre-post non-inferiority trial. It concerned a pilot trial, aimed at examining whether a larger randomized controlled trial of blended versus face-to-face behavioral parent training for ADHD would be justified. Participants were randomly allocated to blended parent training or face-to-face parent training in a 1:1 ratio. Randomization was done by an independent research assistant by means of a random number generator. As this study was a pilot study, a power calculation was not conducted. Researchers, therapists, nor participants were blinded to randomization outcome. The primary parent, i.e. the parent in a two-parent household who spent most time with the child, completed the outcome measures online before randomization and directly after parent training. Parents who did not finish the treatment also completed the post-treatment outcome measures. Children and parents in both conditions were allowed to receive other health care, with the exception of care that resembled the parent training (i.e. behavioral interventions for parents, directed at their child's behavior).

### 2.3 Treatment and therapists

#### 2.3.1 Treatment

The face-to-face and blended parent training addressed the same topics, see Table 1 for an overview of both training formats. The goal of both training formats was to reduce behavior problems in children with ADHD by teaching parents techniques to understand and manipulate their child's behavior. At the beginning of both training programs, parents selected three to five problem behaviors from a list of target behaviors (see Van den Hoofdakker et al., 2007) and three to five situations from the Home Situations Questionnaire (Barkley et al., 1999). Both training formats targeted these specific behaviors and situations.

**Table 1:** Overview of phases and topics in the face-to-face and blended BPT.

Phase	Face-to-face BPT		Blended BPT	
	Session	Description of session	Module	Description of module part
1. Introduction and psycho-education	1	Introduction	1	Introduction (face–to-face)
	2	Psycho education	2	Psycho education
	3	ABC charts	3	ABC charts
	4	Recording observation of behavior		Recording observation of behavior, impeding factors
	5	Analyzing video, impeding factors		
			Evaluation (face-to-face)	
2. Techniques to manipulate antecedents of behavior	6	Communication	4	Communication
	7	Setting rules		Setting rules
	8	Offering structure		Offering structure
			Evaluation (face-to-face)	
3. Techniques to manipulate consequences of behavior	9	Rewarding	5	Rewarding
	10	Ignoring		Ignoring
	11	Punishing		Punishing
			Evaluation (face-to-face)^a^	
	12	Reward system part 1^a^		Reward system part 1^a^
	13	Reward system part 2^a^		Reward system part 2^a^
	14	Reward system part 3^a^		Reward system part 3^a^
	15	Time out^a^		Time out (face-to-face)^a^
4. Evaluation and generalization	16	Evaluation	6	Evaluation (face-to-face)
	Follow up after three months		Follow up after three months (face-to-face)	

a Optional training topics.

The face-to-face parent training program concerned a manualized individual parent training program, which has previously been investigated by Van der Veen-Mulders et al. (2018) and in group format by Van den Hoofdakker et al. (2007). Sessions lasted between 45 and 60 min. At the end of each session parents received homework assignments, which were discussed at the beginning of the next session. The training manual advised the therapists to schedule the first five sessions on a weekly basis and successive sessions weekly or biweekly. Besides the optional sessions (see Table 1), it was advised to add an extra session if parents did not fully understand already discussed topics or experienced difficulties with the techniques of a particular session. The training manual advised therapists to limit the training to a maximum of 17 sessions (excluding the follow up session after three months).

The blended parent training included six online modules with theory and exercises, each consisting of one, two, or three compulsory training parts (see Table 1). The main content of the blended training was provided to parents through the online program. The training started and ended with a clinic-based face-to-face contact. Additionally, the blended training included a few evaluation contacts which were also provided face-to-face. The contact at the start had a duration of 90 min and was used to select target behaviors and situations, as well as to familiarize parents with the online program. The evaluation contacts lasted between 45 and 60 min and concerned the progress of the parents through the program. The blended training manual advised the therapists to give online feedback on each exercise and to provide parents with access to the next training part once the parents had understood the previous content. In addition, the training manual advised therapists to finish the training within 20 weeks and to stimulate parents to work actively in the program by reminding them if there had been no online activity for more than two weeks. If a therapist felt that parents experienced much difficulties with a training part, an extra face-to-face contact could be scheduled to clarify the online content.

#### 2.3.2 Therapists

The face-to-face and blended parent training were delivered by seven therapists from our outpatient mental health clinic. All therapists were licensed psychologists and had several years of experience with behavioral parent training for children with ADHD and behavioral problems. They all had received a two-day training for each parent training format. Therapists who provided the blended parent training had no previous experience with delivering this form of parent training. Psychologists without postmaster education in cognitive behavioral therapy were supervised by a cognitive behavior therapist or a healthcare psychologist formally educated in general behavioral therapy. After randomization of a participant, a therapist was assigned based on availability.

### 2.4 Pretreatment measures

Before randomization, the primary parent provided information on family characteristics and completed the Strengths and Difficulties Questionnaire (SDQ; Van Widenfelt et al., 2003). The Emotional and Peer problems subscales (range 0–10) of the SDQ were used to assess the child's comorbid problems. Psychometric properties of the SDQ have been described as acceptable (Goodman, 2001; Husky et al., 2018; Muris et al., 2003).

### 2.5 Primary outcome measure

The outcome for studying non-inferiority of the blended parent training was the severity of child behavior problems, measured with the Externalizing scale of the Dutch version of the Child Behavior Checklist (CBCL; Achenbach, 1991; Verhulst et al., 1996). Parents indicated whether 33 behaviors did not occur (0), occurred sometimes (1), or occurred often (2) within the last week (i.e. score range 0–66). Reliability and validity of the CBCL are well-established (Achenbach, 1991). Internal consistency was good in the present study (α = 0.88 at both measurement points).

### 2.6 Secondary outcome measures

#### 2.6.1 Attrition

The moment of attrition was defined as the phase of parent training in which parents quit training (i.e. did not start, phase 1, 2, 3, or 4; see Table 1). Furthermore, therapists were asked to rate parental effort by writing down their estimation of ‘the degree of parental involvement/effort’ (range 0–10) after each session (for the face-to-face parent training) or training part (for the blended parent training). Mean parental effort was calculated across the parent training.

#### 2.6.2 Parental satisfaction

The Satisfaction Questionnaire is a self-developed questionnaire based on questions of the Parent Satisfaction Questionnaire (Bearss et al., 2013) and the Therapy Attitude Inventory (Eyberg, 1993; Eyberg and Johnson, 1974). Parents rated 28 questions on a 9-point scale. Subscales concerned 1) the usefulness of the training parts (six questions, e.g. ‘The part about ABC charts was useful’), 2) parent's satisfaction with the therapist (six questions, e.g. ‘The therapist and I got along well’), and 3) their satisfaction with the improvement of their child's behavior (four questions). Other questions include parents' general impression of the training, how easily they could combine the training with their daily life, the degree to which the training had increased their influence on their child's behavior and would do so in the future, the degree to which they used the learned skills, and whether they would recommend the training to other parents. Furthermore, parents were asked which parent training format they had preferred before parent training and which they preferred after parent training. In addition to the Satisfaction Questionnaire, parents indicated how much they agreed with the statement ‘I think the parent training helped to reduce the behavior problems of my child’ (range 1–9).

#### 2.6.3 Therapist time and adherence

We derived the amount of direct therapist time (i.e. face-to-face, online, and telephone therapist-parent contacts) and indirect therapist time (i.e. preparation of sessions and reporting) from the electronic patient records. Time was measured from the first face-to-face parent training session up to the concluding session, or, in case of attrition, up to the moment of discontinuation. When parents did not start the first session, the intended start date was taken as the start of measurement.

Furthermore, therapists' adherence to the treatment manual was measured with a self-developed treatment integrity checklist. Therapists in the face-to-face condition indicated, after each session, whether they had, had not, or had partly addressed each part of a session (e.g. discussing homework with parents, a specific exercise, explaining a new behavioral technique). Therapists in the blended condition did the same for the face-to-face sessions, and indicated whether they had, had not, or had partly given feedback on each online assignment. Adherence was calculated by adding the percentage of fully addressed training parts (i.e. excluding the training parts that could not have been addressed due to parental attrition) to half the percentage of ‘partly’ addressed training parts. Session parts that were addressed in a later session were also included as adherence. In case indication of adherence was missing in over 10% of training parts, the case was excluded for analysis.

### 2.7 Data analysis

Analyses were carried out with IBM SPSS Statistics (v25). Differences in baseline characteristics between the blended and face-to-face condition were analyzed using a Fisher's exac*t*-test (for 2 × 2 tables), Fisher-Freeman-Halton test (for larger tables), independent *t*-test or Mann-Whitney *U* test (exact *p* value), depending on violations of assumptions of normality and equal variances, with a two-sided α of 0.05.

Non-inferiority of the blended parent training, as compared to the face-to-face parent training, was tested with the difference in effect size of the externalizing subscale of the CBCL. This difference was calculated by subtracting the effect size (i.e. [mean CBCL _post_ − mean CBCL _pre_] / *SD*_pre_) of the blended parent training from the effect size of the face-to-face parent training, using a bias correction (Morris, 2008). Pretreatment and posttreatment means and *SDs* were calculated using all available CBCL scores at the corresponding measurement moment. While Morris (2008) advocates pooling the pretreatment *SD*s of the two treatment conditions, we used separate pretreatment *SD*s since the assumption of homogeneous variances was not met. A 90% confidence interval (i.e. using a one sided α of 0.05) was calculated around the difference in effect size, using the posttreatment sample size (see Morris, 2008 for the calculation of the variance). We set a value of 0.12 as the margin of non-inferiority (Blackwelder, 2004). This value was based on two previously found *differences in effect size*. In a meta-analysis by Daley et al. (2014) a value of 0.26 was found for the effect of behavioral treatment (mainly parent training) compared to control groups on conduct problems (i.e. unblinded measures) in children with ADHD. In addition, a value of 0.38 was found on the CBCL (i.e. the same measure as in the current study) in Van den Hoofdakker et al. (2007) for the effect of a group format of face-to-face BPTG compared to routine clinical care in children with ADHD. We considered the difference between 0.26 and 0.38 as a clinically unimportant difference between the face-to-face and blended parent training. Our null hypothesis was that the difference in effect size was 0.12 or higher (i.e. the blended parent training would not be non-inferior to the face-to-face parent training). An upper confidence interval limit lower than 0.12 indicated non-inferiority of the blended parent training (i.e. the alternative hypothesis). The effectiveness of a treatment in a non-inferiority trial (i.e. in this case blended parent training) can be inferred if the effectiveness of the comparison treatment as demonstrated in earlier studies (i.e. in this case face-to-face parent training) is confirmed in the non-inferiority study (Blackwelder, 2004). Therefore, the effect size of the face-to-face parent training was compared to a value of −0.60, which was the effect size found in a previous face-to-face parent training study (Van den Hoofdakker et al., 2007; i.e. recalculated to be comparable), using a 95% confidence interval.

Differences between the two forms of parent training with regard to attrition, parental satisfaction, therapist time, and therapist adherence were explored using a Fisher-Freeman-Halton test, independent *t*-test or Mann-Whitney *U* test (exact *p* value), depending on violations of assumptions of normality and equal variances, with a two-sided α of 0.05.

## 3 Results

### 3.1 Participant flow and baseline characteristics

**Fig. 1:**
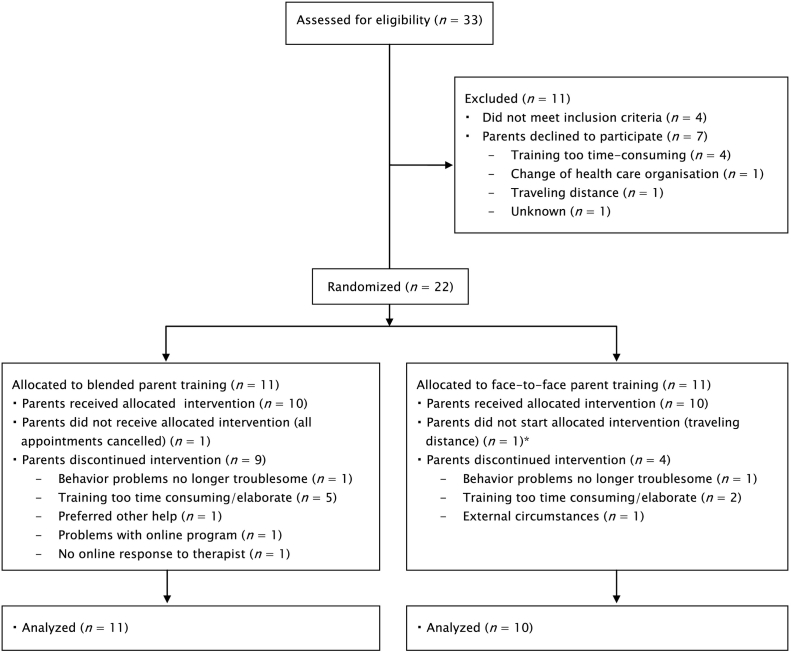
Study's flow chart. *Not included in analyses.

Simultaneous eligibility assessment at the end of the inclusion period led to one more participant than the intended 20. See Fig. 1 for the flow of participants. Baseline characteristics of the blended and face-to-face condition are displayed in Table 2. All primary parents were of Caucasian origin. The treatment groups did not differ on baseline characteristics (Table 2).

**Table 2:** Baseline characteristics of the blended and face-to-face parent training condition and analyses of differences.

	Blended		Face-to-face		Differences between conditions e	*p*
Demographics child a						
Age: mean (*SD*)	7.46	(2.21)	8.10	(1.85)	*U* = 47.5	0.61
Gender: male, *n* (%)	7	(63.6)	8	(80.0)		0.64
IQ: mean (*SD*) b	96.8	(15.5)	96.7	(11.8)	*U* = 40.5	1.00
CBCL at baseline: mean (*SD*) c	21.3	(11.3)	27.1	(2.81)	*t* = 1.65	0.13
Comorbid child problems						
SDQ Emotional: mean (*SD*)	2.73	(1.74)	3.90	(1.79)	*U* = 33.5	0.13
SDQ Peer problems: mean (*SD*)	1.64	(1.63)	2.80	(1.81)	*U* = 43.5	0.15
Comorbid clinical child diagnoses: n (%)					*FFH* = 4.64	0.33
No comorbid diagnoses	7	(63.6)	9	(90)		
Disruptive behavior disorder	2	(18.2)	0	(0)		
Tourette's disorder	1	(9.1)	0	(0)		
Disruptive behavior disorder and Tourette's disorder	1	(9.1)	0	(0)		
Anxiety disorder	0	(0)	1	(10)		
Family characteristics						
Single parent family, *n* (%)	2	(18.1)	4	(40)		0.36
Educational level: *n* (%) d					*FFH* = 2.11	0.45
Low	1	(18.2)	2	(30)		
Middle	6	(54.5)	7	(60)		
High	4	(27.3)	1	(10)		

Note: *n* blended parent training = 11; *n* face-to-face parent training = 10; CBCL: Child Behavior Checklist.

a One child in the blended condition, who met diagnostic criteria for ADHD, had a postponed ADHD diagnosis.

b IQ: *n* blended parent training = 9; *n* face-to-face parent training = 9.

c Range blended parent training = 7–46; range face-to-face parent training = 23–32.

d Concerns the educational level of the parent with the highest educational level in the household. Low = no education, primary school, lower vocational and lower secondary education; middle = intermediate and higher secondary education; high = higher education.

e FFH: Fisher-Freeman-Halton test statistic. Gender and Single parent family were compared between conditions with Fisher's exact test. Since this test does not provide a test statistic, only the *p* value has been reported.

### 3.2 Primary outcome: non-inferiority analysis

The mean posttreatment CBCL Externalizing score was 17.6 in the blended condition (*SD* = 10.5, range = 2–37, *n* = 11) and 19.6 in the face-to-face condition (*SD* = 4.34, range = 14–26, *n* = 8, i.e. two participants did not complete the posttreatment CBCL). The difference in effect size between the two parent training forms was 2.14, with a 90% confidence interval of [0.16, 4.12]. An upper limit of this confidence interval below 0.12 (i.e. the margin of non-inferiority) would have indicated non-inferiority of blended parent training, i.e. we could not reject the null hypothesis (difference in effect size ≥0.12). The effect size of the face-to-face parent training (post – pre) was −2.43 and its confidence interval [−4.73, −0.14] included the effect size of −0.60 that was found in a previous study where face-to-face parent training was found to be effective (Van den Hoofdakker et al., 2007).

### 3.3 Secondary outcomes

#### 3.3.1 Attrition

Table 3 displays the results regarding attrition. All but one participant in the blended condition dropped out before the last phase of the training (90.9%), while this was the case for 40% of the participants in the face-to-face condition. In addition, therapists reported that parents in the blended condition (*M* = 6.81, *SD* = 0.94, *n* = 10) showed less effort during parent training than parents in the face-to-face condition (*M* = 7.78, *SD* = 1.04, *n* = 10; *t* = 2.19, *p* = 0.04).

**Table 3:** Attrition in the blended and face-to-face parent training condition and analysis of differences.

	Blended		Face-to-face		
Phase in which parents stopped	*n*	(%)	*n*	(%)	*p*
Did not start parent training	1	(9.1)	0	(0)	0.01
Phase 1: introduction and psychoeducation	8	(72.7)	1	(10)	
Phase 2: techniques to manipulate antecedents of behavior	1	(9.1)	3	(30)	
Phase 3: techniques to manipulate consequences of behavior	0	(0)	0	(0)	
Phase 4: evaluation and generalization	1	(9.1)	6	(60)	

#### 3.3.2 Parental satisfaction

Table 4 shows the comparison of the blended and face-to-face condition on measures related to parental satisfaction (scale 1–9). Parents in the face-to-face condition were more positive regarding the usefulness of the training parts, their general impression of the training, the increase of their influence on their child, and the reduction of their child's behavior problems, than parents in the blended condition. Furthermore, parents in the face-to-face condition reported that they were more inclined to recommend the training to other parents.

**Table 4:** Parental satisfaction in the blended and face-to-face parent training condition and analyses of differences.

	Blended			Face-to-face			Differences between conditions	*p*
	Mean	(*SD*)	*n*	Mean	(*SD)*	*n*		
Satisfaction Questionnaire subscales								
Usefulness of training parts	7.68	(0.64)	7	8.50	(0.48)	7	*U* = 7	0.03
Satisfaction with therapist	8.62	(0.50)	7	8.96	(0.08)	8	*U* = 16	0.19
Satisfaction with improvement of child's behavior	6.15	(1.37)	10	7.50	(1.13)	8	*U* = 18.5	0.06
Satisfaction Questionnaire items								
General impression of the training	7.43	(1.72)	7	8.88	(0.35)	8	*U* = 10	0.04
Ease of combining training with daily life	4.57	(2.51)	7	6.57	(2.37)	7	*U* = 14	0.21
Training increased influence on behavior child	6.17	(1.84)	6	7.71	(2.98)	7	*U* = 6.5	0.04
Training will increase influence on behavior child in future	6.29	(2.06)	7	7.14	(1.91)	7	*U* = 15.5	0.26
Use of learned training skills	6.57	(0.98)	7	7.50	(1.20)	8	*t* = 1.63	0.13
Recommendation of training to other parents	5.67	(1.97)	6	8.25	(0.71)	8	*U* = 4	0.01
Belief that training helped reduce behavior problems	5.27	(2.24)	11	7.00	(2.00)	7	*U* = 16.5	0.04

Parents in the two conditions did not differ regarding the format they preferred before (*p* = 1.00) and after parent training (*p* = 1.00). At pretreatment, 50% of participants in the blended condition (*n* = 8) and 42.9% of participants in the face-to-face condition (*n* = 7) preferred the face-to-face parent training. At posttreatment, respectively 75% (*n* blended condition = 8) and 87.5% (*n* face-to-face condition = 8) of participants preferred the face-to-face training.

#### 3.3.3 Therapist time and adherence

Therapists in the blended condition spent significantly less direct time (*M* = 493 min, *SD* = 364, *n* = 11) and indirect time (*M* = 268, *SD* = 178, *n* = 11) on parent training than therapists in the face-to-face condition (*M* direct time = 774, *SD* = 226, *n* = 10; *U* = 21, *p* = 0.02 and *M* indirect time = 579, *SD* = 153, *n* = 10; *U* = 12, *p* < 0.01). Regarding adherence to the training manual, therapists in the blended condition (*M* = 86.6%, *SD* = 12.2, *n* = 9) did not differ from therapists in the face-to-face condition (*M* = 93.2%, *SD* = 4.98, *n* = 10; *U* = 29.5, *p* = 0.21). One case (blended condition) was excluded from the therapist adherence analysis due to missing therapist data.

## 4 Discussion

Self-directed parenting programs are on the rise, but it is unknown whether these are a good alternative for therapist-led interventions in referred children with ADHD. The current pilot study aimed to examine the non-inferiority of a blended parent training program in comparison to its face-to-face equivalent in a referred sample of children with ADHD and behavior problems, as well as to investigate whether the blended training differed from the face-to-face training in attrition rates, parental satisfaction, and therapist time. Our study could not demonstrate that blended parent training was non-inferior to face-to-face parent training in the reduction of the child's behavior problems. Furthermore, dropout rates in the blended condition were extremely high and parents in the blended condition dropped out of treatment significantly earlier than parents in the face-to-face condition. Therefore, when interpreting the non-inferiority results, the high dropout rates should be taken into consideration. With regard to parental satisfaction, parents receiving the face-to-face parent training rated the training and its impact as more positive than parents receiving the blended parent training. Finally, therapists in the blended condition spent less time on the training, however, this difference should also be interpreted in light of the higher dropout rate in this condition.

Although we conducted a pilot study with a small sample size, our results regarding the blended parent training stand in contrast with promising findings of other studies on self-directed parent training programs with and without therapist support (e.g. Baumel et al., 2016; Corralejo and Domenech Rodríguez, 2018; Day and Sanders, 2018; DuPaul et al., 2018; Franke et al., 2016; Tarver et al., 2014). However, our study differed in various aspects from most of these other studies. First, we included referred children and compared the self-directed intervention to a therapist-led parent training, instead of to a waitlist control group. Parents of children who are recruited, specifically respond to an invitation to participate in a parent training study. They might therefore be more motivated to complete the training and have more aligned expectations, than parents of children who have been referred to a mental health clinic for behavior problems. Furthermore, parents who choose to participate in a study that only includes self-directed treatment (i.e. as was the case in the studies that have compared this form of treatment to a waitlist control group), may be more motivated to complete the treatment than parents who choose to be randomized to either self-directed or therapist-led parent training. Moreover, parents have been found to rate self-directed parent training less favorable when a therapist-led training is also available (Markie-Dadds and Sanders, 2006). Of note, while all parents at study inclusion indicated they experienced their child to have behavior problems, there may have been less room for improvement in the blended treatment group than in the face-to-face group.

Second, participant characteristics may have differed between our study and other studies. We included children with a clinical diagnosis of ADHD, while other studies into self-directed parent training often selected children using a cut-off on a behavioral problems measure (e.g. studies in the review of Tarver et al., 2014) or DSM ADHD symptom criteria (DuPaul et al., 2018). Referred children with a diagnosis of ADHD and behavioral problems may be more severely disturbed than these children. Although the participants in our study did not appear to have more behavior problems compared to recruited participants (e.g. studies in Tarver et al., 2014), referred children may have more comorbid problems than children recruited from the general population, such as difficulties at school, with siblings, and in the interaction with peers (Coghill et al., 2008; Harpin, 2005). Perhaps, these impairments lead to an increased burden for parents, which make it more difficult for them to complete a self-directed intervention. In addition, parental characteristics may also be different between parents with and without a child with ADHD. For example, since parents of children with an ADHD diagnosis may have more ADHD symptoms themselves, they might have more difficulties with a blended program due to the higher demands on self-discipline (Chronis et al., 2004). In our study, parental effort may have been higher in the face-to-face condition due to the clear expectations of the tasks that parents had to perform each week (Mohr et al., 2011). Furthermore, although our blended training incorporated therapist involvement, our study sample appeared to benefit more when therapist contact was offered permanently, as in the face-to-face parent training. Indeed, it has been suggested that regular therapist-parent contact is a facilitator of effective parenting interventions for ADHD (Smith et al., 2015).

Third, our analysis of behavior problems included study completers and dropouts, while other studies (e.g. studies in Tarver et al., 2014) often reported findings of study completers only. Since attrition rates are high in clinical practice (Chacko et al., 2016), which is once again apparent from the current study, it is important to include dropouts when comparing two types of intervention.

### 4.1 Clinical implications

The possible potential of self-directed parent training with therapist support in addressing attrition in therapist-led parent training for parents of referred children with ADHD is not corroborated by the current results. Therefore, when parents of a child with ADHD in clinical practice express their preference for a self-directed intervention, clinicians need to discuss and clarify the possible limitations of this treatment beforehand, and explain to parents what is expected of them. Furthermore, parents might possibly benefit more from the blended parent training if the training is less flexible, i.e. if the therapist expresses clear expectations of what parents have to do each week, and includes more, and perhaps also more structured, therapist contact. For example, children at risk for ADHD have been shown to benefit from an online parenting intervention in which parents received access to a new part of the online training, as well as a telephone call, on a weekly basis (DuPaul et al., 2018). Future research needs to clarify whether the blended parent training might be a more suitable intervention for other populations, such as parents whose child has not been referred to a mental health clinic or parents of children with other diagnoses than ADHD.

### 4.2 Strengths and limitations

Strengths of our study are its embedment in clinical practice, the inclusion of therapist support in the self-directed intervention, and the inclusion of both treatment completers and non-completers in our analyses. With regard to this last strength, missing CBCL data of two of the four non-completers in the face-to-face condition may have influenced our results in favor of the face-to-face condition.

Our study is limited by its small sample size. Findings should therefore be interpreted with caution. The high dropout rates in the blended condition limit more definite conclusions regarding non-inferiority and amount of therapist time. Furthermore, we did not have enough statistical power to examine possible moderators of treatment effectivity and attrition. In a larger trial, variables such as social economic status, child's age, and level of behavior problems (Baumel et al., 2016; Lundahl et al., 2006) could have shed light on the families that most, or least, benefited from the blended parent training. Another limitation concerns our calculation of the margin of non-inferiority. Ideally, this margin would have been based on the effect size from a previous RCT of the individual face-to-face parent training. Due to absence of such a study, we based it on the results of an RCT on the group format of this training, and additionally, on a meta-analysis of behavioral interventions for children with ADHD. Relatedly, while the current study showed that behavior problems declined over time in the face-to-face parent training condition, our study did not include a care-as-usual or waitlist control group. Therefore, no definite conclusions regarding the effectiveness of the individual face-to-face parent training could be drawn. In addition, due to the small scale and objectives of our pilot study, we did not report on parenting measures (e.g. parenting efficacy, parenting style). Furthermore, we only used parent report measures to assess child behaviors, but no blinded measures. Above mentioned limitations could be addressed in a larger trial, however it is questionable whether our results encourage such a trial. Finally, both parent training programs were to some extent flexible regarding the number of sessions and treatment duration, which may limit the possibility to replicate our findings.

## 5 Conclusion

This head to head comparison between self-directed and therapist-led parent training contributes to our understanding of the value of self-directed parent training with therapist support in clinical practice. Our results showed that a self-directed parent training for parents of referred children with ADHD and behavior problems was not non-inferior to its therapist-led equivalent in reducing children's behavior problems. This result should be interpreted by taking into account the differential levels of attrition in the two treatment conditions as well as the small sample size.

## Role of the funding source

This work was supported by a University Medical Center Groningen (UMCG) healthy ageing pilots grant [grant number 2014-1/189 CDO14.0025]. The UMCG had no involvement in the study design, collection, analysis and interpretation of data, writing of the manuscript, and the decision to submit it for publication.

## Declaration of competing interest

Van den Hoofdakker receives royalties as one of the editors of “Sociaal Onhandig” (published by Van Gorcum), a Dutch book for parents that is being used in the face-to-face behavioral parent training. Non-financial: developed and evaluates several Dutch behavioral training programs for parents, teachers and staff, without financial interests. All other authors have no competing interests.
